# Pain in Rett syndrome: a pilot study and a single case study on the assessment of pain and the construction of a suitable measuring scale

**DOI:** 10.1186/s13023-022-02519-y

**Published:** 2022-09-14

**Authors:** Rosa Angela Fabio, Liliana Chiarini, Virginia Canegallo

**Affiliations:** 1 Department of Economy, University of Messina, via Dei Verdi, 75, 98123 Messina, Italy; 2 Vita-Salute San Raffaele University, Via Olgettina, 58, 20132 Milano, MI Italy; 3 CARI, (Airett Center Innovation and Research), Vicolo Volto S. Luca, 16, 37100 Verona, Italy

**Keywords:** Rett syndrome (RTT), Pain Assessment, Quality of life, Global assessment and intervention Rett scale (GAIRS) Checklist

## Abstract

**Background:**

Rett Syndrome (RTT) is a severe, neurodevelopmental disorder mainly caused by mutations in the MECP2 gene, affecting around 1 in 10,000 female births. Severe physical, language, and social impairments impose a wide range of limitations in the quality of life of the patients with RTT. Comorbidities of patients with RTT are varied and cause a lot of pain, but communicating this suffering is difficult for these patients due to their problems, such as apraxia that does not allow them to express pain in a timely manner, and their difficulties with expressive language that also do not permit them to communicate. Two studies, a pilot study and a single case study, investigate the manifestation of pain of patients with RTT and propose a suitable scale to measure it.

**Aims of this study:**

The first aim was to describe pain situations of RTT by collecting information by parents; the second aim was to test and compare existing questionnaires for non-communicating disorders on pain such as Pain assessment in advanced demenzia (PAINAD), the Critical care pain observation tool (CPOT) and the Non-communicating Children’s Pain Checklist-Revised (NCCPC-R) to assess which of them is best related to the pain behavior of patients with RTT. The third aim was to identify the specific verbal and non-verbal behaviors that characterize pain in girls with Rett syndrome, discriminating them from non-pain behaviors.

**Method:**

Nineteen participants, eighteen girls with RTT and one girl with RTT with 27 manifestations of pain were video-recorded both in pain and base-line conditions. Two independent observers codified the 90 video-recording (36 and 54) to describe their behavioral characteristics.

**Results:**

The two studies showed that the most significant pain behaviors expressed by girls with respect to the baseline condition, at the facial level were a wrinkled forehead, wide eyes, grinding, banging teeth, complaining, making sounds, crying and screaming, and the most common manifestations of the body were tremors, forward and backward movement of the torso, tension in the upper limbs, increased movement of the lower limbs and a sprawling movement affecting the whole body.

**Conclusion:**

The results of the two studies helped to create an easy-to-apply scale that healthcare professionals can use to assess pain in patients with Rett’s syndrome. This scale used PAINAD as its basic structure, with some changes in the items related to the behavior of patients with RTT.

## Background

Rett syndrome (RTT) is a neurodevelopmental disorder that affects approximately 1 in every 10,000 live births, almost exclusively female [1], and is caused by the mutation of a gene in the X chromosome that encodes the binding protein methyl- CpG 2 (MeCP2). Alteration of the MECP2 protein leads to the activation or deactivation of some genes that affect brain development, causing a series of behavioral and neurological alterations [2–4]. The clinical picture is characterized by the progressive loss of manual skills, language, anomalies or absence of movement and by the appearance of stereotypies of the hands, alterations in breathing when awake, which may include hyperventilation and frequent convulsions [5–10]. Various comorbidities are present in RTT in addition to epilepsy, such as gastrointestinal and orthopedic problems, as well as less frequent issues such as endocrinological and cardiac problems, but also liver damage, respiratory disorders, urological dysfunctions, and inflammatory diseases, which make it a very complex and multifaceted syndrome [11–14]. Rett patients may also experience pain caused by other factors such as when confined to a wheel chair, when having routine clinical examinations such as blood draws, etc. According to caregivers, many of these chronic health problems cause pain and impair the quality of life of patients with RTT [12, 15]. Many conditions are particularly painful, such as low bone density. In these individuals, the risk of fractures and microfractures is three to four times greater than in typical individuals [13, 16–18], particularly in the vertebrae and in the femur, thus causing considerable pain. Contractures of the ankle, knees, hip/trunk, elbows and wrist joints have also been reported [19–24]. Less common musculoskeletal problems are also present, such as juvenile idiopathic arthritis [25], osteopenia/osteoporosis [26, 27], joint hypermobility [28–31], muscle atrophy [32], lordosis [33] and torticollis [34, 35]. Skeletal deformations were also found, especially in patients with more severe mutations (e.g. Arg255X) or with large deletions [36–38], especially scoliosis, which, in addition to causing pain, is associated with both an unfavorable prognosis and worse life expectancy [36, 38] and often with severe respiratory tract infections [36]. Breathing difficulties are also among the most common comorbidities. Indeed, during apnea at rest and, less frequently, in hyperventilation, air may be ingested, leading to abdominal swelling, which, in some cases, can lead to gastric perforations and peritonitis [16, 39]. Orally, one of the conditions that causes pain is bruxism, which can eventually lead to tooth wear, fractures, a series of muscle pains in the jaw, and temporomandibular disorders [16, 40–42]. Long-term sucking or biting of the fingers can also lead to mandibular alterations that lead to increased pain [16, 40]. Problems related to the digestive system, such as reflux and esophagitis, are also frequent, and the associated pains worsen or change when lying down [16, 43]. Constipation can also cause pain both in the abdominal area and during the passage of stool [16, 44]. Comorbidities of patients with Rett syndrome are varied and cause a lot of pain, but communicating this suffering is difficult for these patients due to their problems, such as apraxia that does not allow them to express pain in a timely manner [45], and their difficulties with expressive language that also do not permit them to communicate [46–49]. In literature, there have been attempts to identify the modalities of manifestation of pain in patients who have communication difficulties, such as in patients in the final phase of dementia, in advanced malignancy at end-of-life, in severely brain-damaged patients, with a disturbance of consciousness such as coma, a vegetative state or a state in which the subject is unconscious, and mechanically ventilated intensive care unit (ICU) patients [50, 51]. In all these cases, even highly trained physicians, nurses and caregivers may make mistakes in evaluating the presence, location, severity, or impact of pain. Moreover, there are many factors that can influence the underestimation or overestimation of the precise nature of pain, its severity and its location, for example, the subjective nature of pain perception, given that an individual’s threshold experience of pain increases the difficulty of measuring and quantifying the intensity of pain [52].

To try to overcome the problems of subjective measures, various alternative methods for the clinical assessment of pain in non- communicating patients have been proposed such as Pain assessment in advanced dementia (PAINAD) [53], the Critical care pain observation tool (CPOT) [54], the Non-communicating Children’s Pain Checklist-Revised (NCCPC-R) [55], visual analogue scale (VAS), verbal rating scale (VRS), and numerical rating scale (NRS) [56]. McGuire et al. [57] in a study which describes different methodologies, suggest that the physiologic and behavioral dimensions of pain are the most important. Indeed, they can be useful tools that use observable behaviors (such as facial tension or restlessness) to assess pain, and/or physiologic indicators such as vital signs, which are used as cues for more in-depth assessment. For example, the co-associated dimensions of emotional, behavioral and autonomic dysregulation (EBAD) can lead to increases in physical pain and modulation in sensory processing in Rett patients [58]. In patients with RTT, few studies have evaluated pain perception [15, 45, 59]. In the study by O’ Leary et al. [45], scales that take autonomic response into account, such as electrodermal activity (EDA) and heart rate (HR) were used, associated with the Face Legs Activity Cry Consolability (FLACC) behavioral scale [59], which evaluates post-operation pain in young children. Symons et al. [15] described pain sources and frequency using NCCPC-R, which assesses pain in non- communicating children. In the study by Barney et al. (2015) [60], a parent was asked to assess the pain of their daughters through NCCPC-R, the Brief Pain Inventory (BPI) [61] and the Dalhousie Pain Interview (DPI) [55], evaluating pain expression with the Pain Examination Procedure/Pain and Discomfort Scale (PADS/PEP) [62]. BPI is a scale that was originally created for patients with cancer pain, which is now also used with generic pain for other chronic pain conditions and with non-verbal subjects with disabilities [63, 64]. The DPI is used to assess the type, frequency, duration and intensity of pain in children with severe intellectual disability. The PADS/PEP evaluates pain in adults with severe or profound intellectual disability and measures the expression of pain by enabling the evaluator to isolate a source/location of pain [59].

All these scales are generic and do not take the typical characteristics of RTT into account. Patients with Rett syndrome try to communicate their suffering through various behaviors, such as clapping, laughing, delayed pain response, grinding of teeth, sticking out the tongue, moving the body in a specific way, jumping, shaking, self-harming, but also grimacing, vocalizing, moaning, whimpering, screaming, and saying a specific sound or word [15, 16, 38, 59]. The behaviors displayed by subjects with RTT make pain measurement and assessment problematic, especially for healthcare professionals, doctors and nurses who must understand the nature of pain to prescribe and administer drugs.

Since there is no scale in literature that was created exclusively for patients with RTT, the aim of this study is to adapt existing scales to assess pain in patients with Rett syndrome. Many studies rely on voluntary pain induction [65–68], but in this study pain was not induced voluntarily in the girls with RTT and the patients were not hospitalized. Thus, the methodological difficulty was to wait for the spontaneous appearance of pain in RTT patients, not to induce it, and then ask the parents to video-record the event when it occurred.

More in detail, the first aim of the present study is to describe a pain situation by collecting information and by asking the parents the 5 W’s and one H (Who? What? When? Where? Why? How?) questions, in addition to some other questions, such as the estimated intensity of pain, the part of the body with pain, what the parents did to decrease it and the duration of the pain after the intervention of the parents.

The second aim was to test and compare existing questionnaires on pain such as PAINAD [53], CPOT [54], and NCCPC-R [55] to assess which of them is best related to the pain behavior of patients with RTT.

The third aim was to identify the specific verbal and non-verbal behaviors that characterize pain in girls with Rett syndrome, discriminating them from non-pain behaviors. Through analysis of the video-recorded behavioral characteristics, both in the condition of pain and in the condition of baseline (well-being), we tried to identify the most frequent behaviors to be able to discriminate pain from baseline.

Since the pilot study refers to only 18 patients, and since there is wide heterogeneity in Rett syndrome symptoms, we expect wide variability in symptomatology, for this reason we wanted to analyze whether this variability is reduced by analyzing multiple sources (pain situations) from the same patient. To analyze if there are more consistent behaviors in a single patient with respect to more patients, two studies were conducted in the present work: the first refers to a group study and the second to a single case study with repeated measurements of the pain event.

## First study

### Method

#### Patient characteristics

Eighteen female patients diagnosed with RTT, aged between 7 and 29 (M = 17.98, SD = 6.63; 100% Caucasian) were recruited by the Associazione Italiana Rett (AIRETT). The participants received a pre-intervention global assessment which included two scales: The Rett Assessment Rating Scales (RARS) [69] and the GAIRS Checklist [70–72] that were used to assess severity and functioning.

Table 1 shows the descriptive characteristics of the patients.

**Table 1:** Participant characteristics

Participant	Age	Mutation	RARS	GAIRS
1	21	c.880 C > T, p. Arg 309 Trp	63.00	141.00
2	13	c.806delG	55.00	183.00
3	29	R306C	77.00	167.00
4	29	R294X	62.00	180.23
5	7	R270X	70.00	167.00
6	13	c.806delG	70.00	165.91
7	25	mcp2 270arg stop	63.00	182.22
8	16	PCDH19	61.00	184.09
9	24	P152R	47.5	176.00
10	24	P152R	49.12	176.00
11	8	R306C	55.00	192.03
12	21	R294X	77.00	155.00
13	21	R270X	68.00	176.00
14	23	c.806delG	70.00	178.09
15	18	R270X	70.00	176.09
16	22	R255	55.00	198.23
17	19	c.880 C > T, p. Arg 309 Trp	81.00	149.00
18	17	c.880 C > T, p. Arg 309 Trp	70.00	156.90

Appropriate ethical approval was obtained for this study and informed consent was obtained on behalf of all individuals included in the study (University of Messina protocol number: 2020/33). The parents also signed the Video Recording Consent Form according to the European Environment Agency (EEA) suggestions.

#### Parent questionnaire

Parents were asked, if over a month, their child had an episode of pain, to video-record the episode for one or two minutes, focusing on the face and on the arms and legs. After the pain episode, they were also asked to fill in a brief questionnaire. The questionnaire completed by the parents provides information on the condition which the patient was in before the video-recorded pain episode. Table 2 shows the questions the parents were asked. In order to have a baseline condition to compare behaviors with and without pain, the parents were asked to video-record also an episode of calm or pleasure and to reply to the related questions.

**Table 2:** a PAIN. Parent questionnaire. b Baseline parent questionnaire

*a*
Today is:
1. When did it manifest? (date and time)
2. How long did it last?
3. What was the child doing before she was in pain and where was she?
4. Which behavior did your daughter manifest that made you realize she was sick?
5. According to your daughter, where was the pain (belly, teeth, head…)?
6. If you were to write on a scale from 1 (minimum pain) to 10 (maximum), how intense was it?
7. What did you do to ease the pain?
8. When you intervened, how long did the intervention last?
*b*
Date:
1.When did it manifest? (date and time)
2. How long did it last?
3. What was the child doing before the pain started and where was she?
4. What behavior did your daughter manifest to show you she was happy?
5. According to her what makes her feel good?
6. If you were to write on a scale from 1 (minimum pleasure) to 10 (maximum), how intense was it?

The questionnaire completed in the moment of pleasure consists of six questions, which invited the parent to indicate when the moment of well-being occurred, how long it lasted, what the girl was doing previously, where she was, what behavior made it clear that her daughter was happy, what made her feel good and, finally, the intensity from 1 (minimum pleasure) to 10 (maximum pleasure). The questionnaire completed in the moment of pain was characterized by eight questions that invited the parent to indicate when the pain occurred, how long it lasted, what their daughter was doing previously, where she was, what the behavior was that made it clear that their daughter was sick, the type of pain their daughter felt according to the parent, the intensity on a scale of 1 (minimum pain) to 10 (maximum pain), what was done to relieve it, and finally, how long it was before the pain subsided.

#### Observer evaluation of video-recorded event

Each independent observer evaluated the pain episode and the calm episode for each girl, three times: with PAINAD, CPOT and NCCPC-R scales.

The PAINAD scale [53] is an observational scale for patients with cognitive deficits, used with patients with dementia. It includes five behavioral subscales such as: breath, vocalization, facial expression, body language and comfort. Each of these is assigned a score ranging from 0 to 2 in increasing order of discomfort. The sum of the individual scores results in a pain value that can range from 0 to 10. Initial PAINAD assessments were performed in two studies, both in the Veterans Health Administration’s long-term dementia special care units [53]. Internal consistency was assessed based on a pooled sample from both studies. Cronbach’s alpha in three situations ranged from 0.50 to 0.65 [53]. Pearson’s correlation coefficient during pleasant activity was 0.97 and during unpleasant activity 0.82 [53, 71]. Inter-rater reliability was strong in five studies [53, 73].

CPOT is a scale created by Gelinas et al. [54]. This tool is used in intensive care for subjects who are unable to communicate. It is characterized by four domains which include: facial expression, body movements, muscle tension and compliance with ventilation or vocalization. Each of the four areas is rated from 0 to 2 with a total score ranging from 0 to 8, where 0 represents no pain, and 8 the maximum pain. The domain concerning compliance with ventilation or vocalization was not considered in our study. CPOT has good psychometric indices regarding the inter-observer agreement of assessments in medical patients and surgeons [74–76]. CPOT has demonstrated inter-rater reliability with coefficients k between 0.52 and 0.80 [74–76]. The content validity of CPOT was ascertained by four doctors and thirteen intensive care nurses [74–76].

NCCPC-R is a checklist for children who are unable to speak due to physical or/and cognitive disabilities [55]. This scale was designed for untrained parents and caregivers, but also for adults who are unfamiliar with children with these disabilities [55]. It is characterized by six domains: vocal expression (4 items), sociability (4 items), face (5 items), motor activity (2 items), body and limbs (6 items), physiological state (5 items), and finally the domain concerning “eating/sleeping” (3 items). In this study, the latter subscale was not applied. For each item, the score ranges from 0 (not applicable) to 3 (very often), in our study 0 was interpreted as absent, 1 as only a little, 2 strong, 3 very strong. In the Italian validation, high values were found regarding the intra and interclass correlation coefficient (ICC), which indicates high reliability, together with the high value of Cronbach’s alpha coefficient, indicating high validity [77].

#### Procedure

The survey was conducted from May 2021 to November 2021. Each parent was required to observe the behaviors of their daughter when she exhibited both pain and well-being or calm episodes. When one of the two situations occurred, the parent had to video-record their daughter with their mobile phone, framing her face and limbs, making a video lasting from 1 to 3 min, to capture all the expressions and behaviors of their daughter. For each video, the parent was also asked to fill in a questionnaire (Table 2) that allowed them to better understand the situation their daughter was in.

The videos, with the attached questionnaires, were sent to an e-mail address for review. Considering the associated questionnaire, each video was observed and measured with three rating scales: the PAINAD, the CPOT and the NCCPC-R scales.

Forty percent of the video protocols were analyzed by 5 independent researchers on the 3 scales. Observer agreement rate was 95% and differences in agreement were discussed and resolved. The remaining protocols were independently examined by two researchers and observer agreement was over 98%.

In addition to the analysis of the coded scales that already exist in literature, detailed decoding of second-by-second behaviors produced during the pain phase and the baseline phase (videos of patients in conditions of calm or pleasure) was carried out by two other independent researchers (Cohen’s k = 0.98). They had to codify each movement of the body referred to the head (eyes, mouth, forehead, sounds of the mouth), to the central part of the body (shoulders, arms, hands, back, stomach) and to the lower part of the body (legs, knees, feet), second-by-second.

### Results

Data were analyzed using the Statistical Package for the Social Sciences, version 25. Means and standard deviations (SD) for the descriptive variables were used. Normality of the distributions of quantitative variables was verified by applying the Shapiro–Wilk test. Descriptive analysis of both demographic and clinical characteristics of Rett Syndrome patients was performed on the entire cohort. Results were discussed initially with reference to the parents’ questionnaires, secondly, by examination of the existing pain scales and finally, an analysis of video-recorded behavioral characteristics of pain in patients with Rett syndrome.

#### Parents questionnaire

Table 3 show the questions the parents were asked and their main replies.

**Table 3:** Main category of parents’ replies

Questions			First study	Second study
When did it manifest paint?	Afternoon		80%	60%
	Morning		20%	40%
Where was the child before she felt pain?	Home		90%	84%
	Outside		10%	16%
What the was child doing before she was in pain?	Static activities		68%	68%
		Listening to music		16%
		Being in the car		4%
		Outdoor in a wheelchair	12%	8%
		Eating	6%	20%
		Watching TV	18%	16%
		Taking a lesson	6%	4%
		Sleeping	12%	
		Sitting in the garden	12%	
	Dynamic activities		31%	32%
		getting ready/dressing	6%	20%
		Doing exercises		4%
		Playing	12%	8%
		Walking	12%	
According to your daughter, where was the pain?		Intestines or belly	61%	72%
		Uterus	25%	26%
		Seizures	6%	16%
		Disorders of various kinds	8%	
		Experience of discomfort		8%
What did you do to ease the pain?	Natural interventions		55%	56%
		Massage	31%	4%
		Giving food/water	6%	40%
		Talking		16%
		Listening to music	6%	12%
		Loosening the seat belt		12%
		Cuddle	6%	
	Pharmacological interventions		45%	44%
		Anti- inflammatory	33%	36%
		Painkiller patch		8%
		Blower	6%	
		Supplements	6%	
	Combined interventions		16%	16%
Average pain intensity (scale ranging from 1 to 10)			8.36	7.94

As can be seen, most patients (80%) experienced pain in the afternoon, and only a small percentage (20%) in the morning. The place where the pain was more manifested was the home (90%), compared to outside (10%). Thirty-eight percent of them were doing static activities when the pain manifested, such as watching TV, eating, sitting in a wheelchair, sitting in the garden, 11% were sleeping, and 38% were doing dynamic activities such as walking, dressing or standing. As referred by the parents, 61% of the patients experienced intestinal or stomach-related problems, 25% pain in the uterus, 6% had convulsions, and 8% disorders of various kinds.

The average pain intensity was 7.94 (with a scale ranging from 1 to 10; SD = 2.10). Twenty-six percent of the parents massaged the patients to alleviate the pain and the pain decreased after 15 min, 33% administered anti-inflammatories (paracetamol, ibuprofen). Average time of pain decrease was 180 min, in interventions related to evacuation the average time to pain relief was 40 min. In our protocols, all interventions in which intensity was equal to 3–6, whose average duration was 43 min and which were resolved only with comforting or massages were evaluated as discomfort (not pain) and excluded.

#### Analysis of the pain scales

Table 4 shows mean and standard deviations of the PAINAD, CPOT and NCCPC-R scales and their subscales both in pain and baseline conditions.

**Table 4:** Mean (and Standard Deviation) of the PAINAD, CPOT and NCCPC-R scales in pain and baseline conditions

Scales	Pain setting	Baseline setting
Painad total results	8.00 (1.24)	0.60 (0.65)
Painad breath results	1.38 (0.55)	0.40(0.65)
Painad vocalization results	1.44 (0.74)	0.20 (0.42)
Painad facial expression results	1.86 (0.47)	0.00 (0.00)
Painad body language results	1.52 (0.39)	0.00 (0.00)
Painad consolation results	1.52 (0.67)	0.00 (0.00)
CPOT total results	5.25 (1.08)	0.20 (0.47)
CPOT facial expression results	1.80 (0.38)	0.00 (0.00)
CPOT body movement results	1.67 (0.45)	0.20 (0.63)
CPOT muscle tension results	1.78 (0.39)	0.00 (0.00)
NCCPC-R total results	32.83 (15.33)	1.25 (1.13)
NCCPC-R vocal results	4.78 (3.37)	0.10 (0.31)
NCCPC-R social results	6.83 (1.79)	0.00 (0.00)
NCCPC-R facial results	9.00 (4.03)	0.35 (0.74)
NCCPC-R activity and body/ limb results	6.77 (2.77)	0.40 (0.96)
NCCPC-R physiological sign results	5.44 (2.51)	0.40 (0.69)

Paired t-tests were applied to compare differences between the baseline condition and the pain condition and Pearson correlations were applied to study the relationship between the three pain scales.

#### Paired t-tests

From this analysis, significant differences were found in relation to PAINAD, t (17) = 26.36, *p* < 0.001, CPOT, t (17) = 19.66 *p* < 0.001, and NCCPC-P, t (17) = 15.66 *p* < 0.001. Therefore, all 3 scales are able to discriminate the presence and the absence of pain.

#### Pearson correlations

Analysis of the relationship between the three pain scales shows that PAINAD and CPOT are strongly correlated, r (18) = 0.53, *p* < 0.02, while NCCPC-R is weakly correlated with both PAINAD and CPOT, respectively r (18) = 0.32, *p* = 0.18, and r (18) = 0.36, *p* = 0.07. Moreover, the subscales of NCCPC-R have no significant correlations with the total PAINAD scales and CPOT. Moreover, PAINAD was significantly correlated with intensity of pain of the judgment of parents (r = 0.55, p < 0.01).

#### Analysis of video-recorded behavioral characteristics of pain

The third aim was to identify the specific verbal and non-verbal behaviors that characterize pain in girls with Rett syndrome, discriminating it from discomfort or boredom. Two observers, separately and independently, carried out the second-by-second codifying of all the video-recorded sessions. Each observer had to classify the specific behaviors related to parts of the body (face, limbs, torso) and to social interaction seen in the patient, for example: “moves the tongue”, “moves the arms”, “withdraws when someone try to interact”. A total of 36 videos referred to 18 patients were examined: 18 referred to baseline condition and 18 to pain condition. The inter-rater reliability for categorical behaviors, using Kendall’s coefficient of concordance (Wa) [78] was very high (k = 0.98).

From the second-by-second behavioral analysis of the videos, it emerges that, in the pain setting, the patients had a wide range of behavioral modifications compared to the baseline setting.

**Fig. 1:**
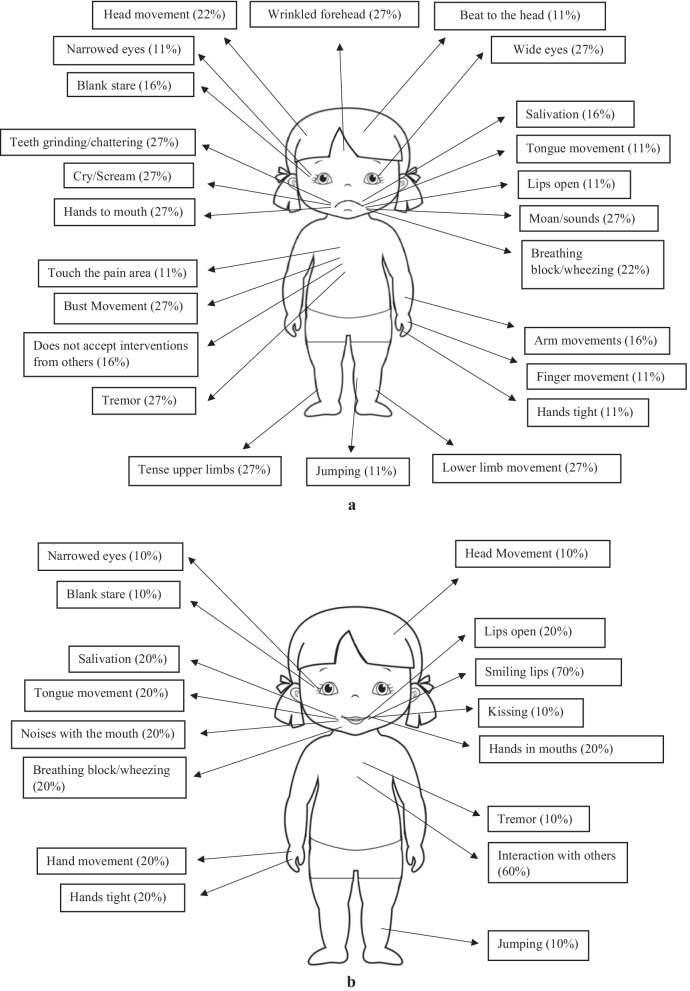
**a** Manifestation of behaviors derived from observations in moments of pain of the 18 girls. **b** Manifestation of behaviors derived from the observations of moments of baseline of the 18 girls

Figures 1a and b shows the related percentages of the specific behaviors in both pain and baseline settings.

The data show that the most significant factors of pain expressed by girls compared to the baseline, at the facial level, are related to a wrinkled forehead (χ^2^ (17) = 15.125, *p* < 0.01), wide eyes (χ^2^ (17) = 15.125, *p* < 0.01, grinding and banging teeth (χ^2^ (17) = 14.82, *p* < 0.01), making moans and sounds (χ^2^ (17) = 12.45, *p* < 0.01), crying and screaming(χ^2^ (17) = 15.125, *p* < 0.01); behavioral manifestations regarding the body include tremors (χ^2^ (17) = 15.125, *p* < 0.01), forward and backward movement of the torso (χ^2^ (17) = 13.33, *p* < 0.01), tension in the upper limbs (χ^2^ (17) = 16.125, *p* < 0.01) and increased movement of the lower limbs (χ^2^ (17) = 16.34, *p* < 0.01). In baseline condition, in which pain is absent, it emerges that the girls smile more (χ^2^ (17) = 19.33, *p* < 0.01) and interact more (χ^2^ (17) = 19.33, *p* < 0.01).

In this study, only one patient did not have severe physical apraxia and could gesticulate to communicate the place of pain clearly, and only 1 girl, experiencing menstrual pain, implemented behaviors that let the observer guess the area of pain, such as putting her hands on her hips and bending forward with her torso: therefore, the objective of understanding what part of the body is painful could not be met.

## Second study

The method related to the second study is the same as the first, but refers to 27 pain episodes, in the same period of time (May 2021-November 2021), of only one girl with Rett syndrome. The patient is 18 years old, has a R255 mutation, a RARS global score of 61 and a GAIRS score of 191.

### Results of second study

Again, firstly, results related to the parents’ questionnaire are presented, secondly, the relationship between the existing pain scales and, finally, the analysis of 27 video-recorded behavioral characteristics of pain of the same patient.

#### Parent questionnaire

Table 3 shows the questions the parents were asked and their main replies.

The patient experienced pain more often in the afternoon (60%) and less in the morning (40%). The place where the pain was more manifested was again in the home (86%), compared to outside (10%). Most of the times the girl was performing static activities (68%), such as listening to music (16%), being in the car (4%), being taken for a walk in a wheelchair (8%), eating (20%), watching TV (16%), taking lessons (4%), and only a few times was she performing dynamic activities (32%) such as getting ready/dressing (20%), doing exercises (4%) and playing (8%). Average pain intensity was 8.36 (SD = 1.89). The pain involved the intestines or abdomen (72%), the uterus (26%), was accompanied by seizures (16%) and, only a few times, discomfort (8%). In 56% of the time, only natural interventions were applied and the average duration of pain was 22.5 min, 28% of the time interventions were implemented through drugs and the pain subsided, on average, after 27.86 min, 16% of the time combined interventions were implemented and, on average, after 28.75 min the pain was relieved. The natural interventions involved giving food/water (40%), talking (16%), doing massages (4%), listening to music (12%) and loosening the seat belt (12%), while pharmacological interventions involved the administration of paracetamol (28%), ibuprofen (8%) and painkiller patch (8%).

#### Analysis of the pain scales

Table 5 shows mean and standard deviations of the PAINAD, CPOT and NCCPC-R scales and their subscales both in pain and baseline conditions.

**Table 5:** Mean (and Standard Deviation) of the PAINAD, CPOT and NCCPC-R scales in pain and baseline conditions

Scales	Pain setting	Baseline setting
Painad total results	8.28 (1.60)	1.00 (0.00)
Painad breath results	1.66 (0.45)	1.00 (0.00)
Painad vocalization results	1.52 (0.71)	0.00 (0.00)
Painad facial expression results	1.62 (0.46)	0.00 (0.00)
Painad body language results	1.80 (0.40)	0.00 (0.00)
Painad consolation results	1.68 (0.47)	0.00 (0.00)
CPOT total results	5.10 (1.19)	0.00 (0.00)
CPOT facial expression results	1.70 (0.52)	0.00 (0.00)
CPOT body movement results	1.84 (0.37)	0.00 (0.00)
CPOT muscle tension results	1.56 (0.65)	0.00 (0.00)
NCCPC-R total results	31.36 (7.74)	1.75 (0.35)
NCCPC-R vocal results	4.68 (3.02)	0.00 (0.00)
NCCPC-R social results	6.70 (2.00)	0.00 (0.00)
NCCPC-R facial results	10.02 (3.12)	0.75 (1.06)
NCCPC-R activity and body/ limb results	5.48 (1.32)	0.00 (0.00)
NCCPC-R physiological sign results	4.48 (2.46)	1.00 (1.41)

Table 5 Mean (and standard deviation) of the PAINAD, CPOT and NCCPC-R scales in pain and baseline conditions.

Paired t-tests were applied to compare differences between the baseline condition and the pain condition and correlational analysis to analyze the relationship between the three scales.

#### Paired t-test

From this analysis, significant differences were found in relation to PAINAD, t (26) = 6. 53, *p* < 0.001, CPOT, t (26) = 9.76 *p* < 0.001, and NCCPC-P, t (26) = 10.08 *p* < 0.001. Therefore, all 3 scales were able to discriminate the presence and the absence of pain.

#### Pearson correlation

Analysis of the relationship between the three pain scales shows that PAINAD and CPOT are strongly correlated, r (26) = 0.54, *p* < 0.01, NCCPC-R is also significantly correlated with both PAINAD and CPOT, respectively r (26) = 0.66, *p* < 0.01, r (26) = 0.50 *p* < 0.01; PAINAD was significantly related to the judgment of intensity of the parents (r = 0.54, *p* < 0.01).

#### Analysis of video-recorded behavioral characteristics of pain

The third aim was to identify the specific verbal and non-verbal behaviors that characterize pain in the girl with Rett syndrome who had 27 pain episodes. Two observers, separately and independently, carried out the codifying of all the video-recorded sessions. Each observer classified the specific behaviors related to different parts of the body. A total of 45 videos were examined: 18 referred to baseline condition and 27 to pain condition. The inter-rater reliability for categorical behaviors, using Cohen’s K coefficient of concordance was very high (k = 0.95).

From the behavioral analysis of the videos, it emerges that, in the pain setting, also this patient had a wide range of behavioral modifications with respect to the baseline setting.

**Fig. 2:**
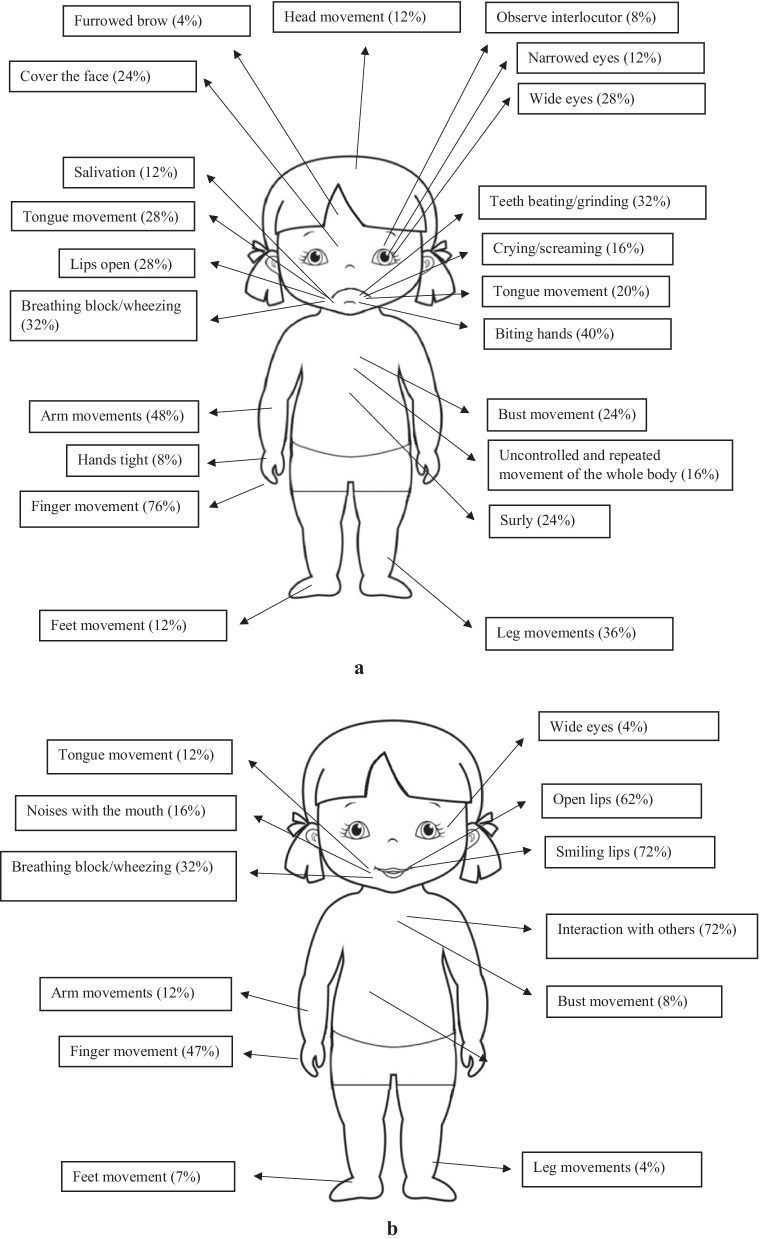
**a** Manifestation of behaviors derived from observation of moments of pain of the single case. **b** Manifestation of behaviors derived from observation of moments of well-being of the single case

Figures 2a and b show the related percentages of the specific behaviors in both pain and baseline settings.

As shown in Fig. 2, unlike the study of the 18 girls, this study relating to a single subject shows that the specific behaviors of pain tend to aggregate around 6 areas of the body and the frequencies were higher. Compared to the previous study, the girl showed movement anomalies (χ^2^ (26) = 18.82, *p* < 0.01 and repetitive dystonic postures affecting the whole body (χ^2^ (26) = 20.09, *p* < 0.01 and this happened both when she was in pain and when she faced strong emotions. In baseline condition, in which pain is absent, it emerges that the girl smiles more (χ^2^ (26) = 22.6, *p* < 0.01) and interacts more (χ^2^ (26) = 21.87, *p* < 0.01).

## Discussion

This study had the main objective of adapting and proposing a new easy-to-apply scale with a suitable measurement for the evaluation of pain in patients with Rett syndrome. In this research, two studies were carried out: one consisting of 18 patients (study 1) and one of a single case study (study 2) that showed 27 pain episodes in the same period of time in which the first was administered. In both studies, we used NCCPC-R [55], PAINAD [53], and the CPOT scales [54]. The three scales were compared with the condition of pain and the base-line condition and it emerged that all 3 scales were able to discriminate the presence of pain, compared to the absence of pain. We chose PAINAD as it had the highest levels of correlation with the judgment of parents and a high level of significance in discriminating between the presence and absence of pain [15].

The two studies showed that the most significant pain behaviors expressed by girls with respect to the baseline condition, at the facial level were a wrinkled forehead, wide eyes, grinding, banging teeth, complaining, making sounds, crying and screaming, and the most common manifestations of the body were tremors, forward and backward movement of the torso, tension in the upper limbs, increased movement of the lower limbs and a sprawling movement affecting the whole body.

In both studies, there were few videos related to the annoyance state, therefore, it was not possible to discriminate between pain vs annoyance. Results related to the manifestations of pain are in agreement with various studies [15, 17], in which it was noted that the girls communicated pain through facial expressions, vocalizations, laments, screams, cries, grinding teeth, moving their tongue, jumping, shaking, altered breathing but also groaning, saying a specific word, stiffening, tearing and changing color.

In our study, there was only one patient without apraxia, for this reason we were unable to exactly identify the area of pain.

The behaviors manifested in both studies were taken into consideration when creating a scale with the typical manifestations of patients with Rett syndrome. By correlating the observations of behaviors and the scales used it was decided to adopt the basic structure of PAINAD [53], as it is more consistent with the behaviors emitted by patients with Rett syndrome. Moreover, since in the original version of PAINAD there were few references to specific behaviors of patients with RTT, we modified some items of the PAINAD scale: in the area of “vocalizations” at score 1, we have added “grinding teeth”, to score 2, the item “repeated calls” has been eliminated, as it is not present in the typical behaviors of RTT girls, and has been replaced with “Scream”; in the subscale “facial expression” to indicate score 2, the “wide eyes”, the “wrinkled forehead” and finally, in the sub-area of “language of the body”, the items “tremor”, “rocking” and “biting the hands” were added to score 2.

The final scale called PAINAD-RTT that this study is proposing is illustrated in Table 6.

**Table 6:** PAINAD-RTT

	0	1	2
Breath (Independent of vocalization)	Normal	Breathing at times altered Short periods of hyperventilation	Impaired breathing Hyperventilation Cheyne- Stokes
Vocalization	None	Occasional moans Occasional negative expressions Grinding of teeth	Complaints Cry Scream
Facial expression	Smiling or Expressionless	Sad Anxious Contract	Grimaces Wide eyes Wrinkled forehead
Body language	Relaxed	Tense. Nervous movements. Restlessness	Rigidity. Agitation Knees bent. A finalistic jerky movement. Tremor. Rocking. Biting hands
Consolability	Does not require consolation	Distracted or reassured by voice or touch	Inconsolable, is neither distracted nor reassured
TOTAL			

### Limits and future prospects

Since the sample on which the study was carried out is limited, the behaviors manifested in moments of pain identified and inserted in the scale may not cover all the typical manifestations of Rett syndrome, which with a larger sample it would be possible to provide a much broader overview. In addition, it was not possible to take into account behaviors related to annoyance as there were few videos, and there were no videos related to boredom. Again, in future studies, a larger sample could be used in different situations of pain, discomfort and boredom to better discriminate the various states. In addition, health care staff might be asked to identify various behaviors as girls experience pain within hospital settings. This would be an aid in assessing agreement with parents and investigating what areas health care staff should consider when assessing pain.

Moreover, another limitation is related to the scales used in this work. For example, the Pain Assessment in Advanced Dementia Scale (PAINAD) scale, although it might seem to be consistent with behaviors exhibited by Rett patients, it is a scale for dementia so the manifestations of pain captured by PAINAD are probably different in patients with Rett syndrome and in patients with dementia. As known, Rett syndrome is not a neurodegenerative disorder, for this reason it is important to understand that one must have this caution in interpreting the symptoms.

Moreover, based on the mutations of the MECP2 gene, we know that there are different responses to painful stimuli [79, 80]: in the present study, only patients with a limited type of specific mutations were included.

## Conclusion

The results of the two studies have helped create a scale that healthcare professionals can use to assess pain in patients with Rett’s syndrome. The scale used PAINAD as its basic structure, with some changes in the items related to the behavior of patients with RTT. However, the sample on which the study was carried out is small, and thus the behaviors manifested in moments of pain that were identified and included in the scale may not cover all the typical manifestations of Rett syndrome, which a larger sample might have encountered. Therefore, in the future, a larger sample with more situations of pain, discomfort and boredom could be used to better discriminate the various manifestations.

## Data Availability

The data can be obtained from the corresponding author upon request.

## Abbreviations

RTT: Rett syndrome
GAIRS: Global assessment and intervention in Rett syndrome
CPOT: Critical care pain observation tool
NCCPC-R: Non-communicating children’s pain checklist-revised
NRS: Numerical rating scale
PAINAD: Pain assessment in advanced dementia
RARS: Rett assessment rating scales
VAS: Visual analogue scale
VRS: Verbal rating scale
AIRETT: Italian Rett association
